# Presentation, diagnosis and management of locally advanced breast cancer: Is it different in low/middle income countries?

**DOI:** 10.12669/pjms.35.6.165

**Published:** 2019

**Authors:** Shahneela Manzoor, Mariyah Anwer, Salim Soomro, Dileep Kumar

**Affiliations:** 1 Dr. Shahneela Manzoor, MBBS, FCPS Trainee (General Surgery). Jinnah Postgraduate Medical Center, Karachi, Pakistan; 2 Dr. Mariyah Anwer, MBBS, FCPS. Jinnah Postgraduate Medical Center, Karachi, Pakistan Assistant Professor of Surgery, Jinnah Postgraduate Medical Center, Karachi, Pakistan; 3 Dr. Salim Ahmed Soomro, MBBS, FCPS. Professor of Surgery, Jinnah Postgraduate Medical Center, Karachi, Pakistan; 4 Dr. Dileep Kumar, MBBS, FCPS. Associate Professor of Surgery, Jinnah Postgraduate Medical Center, Karachi, Pakistan

**Keywords:** Breast Cancer, Third-World Countries

## Abstract

**Background and Objective:**

Breast cancer incidence is increasing and it is highest in low income countries. The main challenge is regarding awareness, screening, late presentation and its management in a third-world country. Our objective was to share the experience faced in various presentations, diagnosis and management of locally advanced breast cancer (LABC) in a third world country and discuss if they are different with respect to developed countries.

**Methods:**

It is a retrospective case series study performed at Jinnah Postgraduate Medical Centre Karachi, from January 2015 to December 2017, over period of three years. Data was collected from the record maintained by authors about patients presenting in breast clinic. Patients of breast cancer were managed in breast clinic over 3 years. Our study included patients who presented with LABC based on their clinical presentation confirmed by histopathological diagnosis and followed by surgical management. Statistical analysis for students t-test was performed using SPSS (version 20.0). A p-value less than 0.05 was considered statistically significant.

**Results:**

One hundred twelve patients presented with LABC over period of 3 years. All but two were female. Mean age was 52 years (range 26-78 years), SD 18.96. On presentation size of tumor was 5cm & more in 103 (91.9%) patients. Involvement of axilla was in 86 (76.7%). Chest wall was involved in 22 (19.6%). Total T3 and T4 were 71 (63.3%) and 41 (36.6%) respectively Diagnosis of all patients was confirmed by histopathology. Neoadjuvant was given to all patients to downstage the tumor. ER/PR was positive in 46(41.1%), HER-2/neu positive in 31 (27.6%). On staging breast carcinoma was metastatic in 13(11.6%) with liver, lung and bone in 4 (3.5%), 3 (2.7%) and 6 (5.3%) respectively. Breast conservation was done in 6 (6.1%) patients, Modified Radical Mastectomy was done in 86 (86.9%), Radical Mastectomy in 3 (3.03%), Toilet Mastectomy in 4 (4.045) and 13 (11.6%) patients were not operated.

**Conclusion:**

In our series 65% of all breast cancers are LABC at presentation. In low/middle income countries high percentage of LABC at presentation result in high metastatic disease, poor prognosis and limits conservation of breast. Awareness and education can improve outcomes.

## INTRODUCTION

Breast cancer in LMICs often presents with locally advanced breast cancer (LABC).^1^ LABC accounts for 40-60% of all breast cancers on presentation in developing countries.^2^ One of the most important prognostic factors of survival in breast cancer is the clinical stage at diagnosis.^3^ Since 2008, breast cancer incidence has increased by over 20% and breast cancer deaths have risen by 14%.^4^ Breast cancer is the commonest female malignancy all over the world including Pakistan and a 2nd leading cause of death from cancer in female population due to late presentation and advance stage of disease.^5^

The majority of women present with advanced disease stage III and IV and the 5-year survival rate in less than 50%.^3^ Advance breast cancer and its high mortality are seen with delay in diagnosis and treatment.^6^ Third world breast cancer is characterized by late presentation, advance stage of disease with a worse biologic behavior and occurrence relatively at a younger age than that reported in western literature.^7^ Our aim of study was to share the experience faced in various presentations, diagnosis and management of locally advanced breast cancer (LABC) in a third world country and discuss if they are different with respect to developed countries.

## METHODS

It is a retrospective case series study done in surgical ward of a teaching hospital Jinnah Postgraduate Medical Center in Karachi Pakistan, in three years from January 2015 to December 2017. Data was collected from the record maintained by authors about patients presenting in breast clinic. Total 172 patients of breast cancer were managed in breast clinic over three years. Patients admitted in surgical ward from surgical OPD were enrolled in the study after taking informed consent. Our study included 112 patients i.e. 65% of total patients, who presented with LABC based on their clinical presentation confirmed by histopathological diagnosis and followed by surgical management. Patients of early breast cancer were excluded.

Statistical analysis for students t-test was performed using SPSS (version 20.0). A p-value less than 0.05 was considered statistically significant. The study was approved by the JPMC Ethics Committee (F2-81-GENL/JPMC).

## RESULTS

**Fig. 1:**
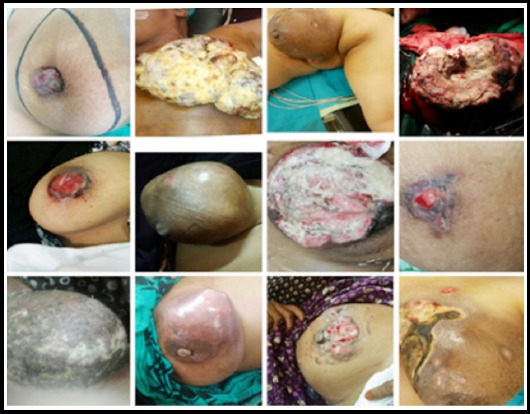
Showing late presentations of breast cancer.

One hundred twelve patients presented with LABC over period of three years. All but two were female. Mean age was 52 years (range 26-78 years), SD 18.96. On presentation size of tumor was 5cm & more in 103 (91.9%) patients. Involvement of axilla was in 86 (76.7%). Chest wall was involved in 22 (19.6%). Total T3 and T4 were 71 (63.3%) and 41 (36.6%) respectively (Fig. 1). Diagnosis of all patients was confirmed by histopathology. Neoadjuvant was given to all patients to downstage the tumor. ER/PR was positive in 46(41.1%), HER-2/neu positive in 31 (27.6%). On staging breast carcinoma was metastatic in 13(11.6%) with liver, lung and bone in 4 (3.5%), 3 (2.7%) and 6 (5.3%) respectively (Table-I). Breast conservation was done in 6 (6.1%) patients, Modified Radical Mastectomy was done in 86 (86.9%), Radical Mastectomy in 3 (3.03%), Toilet Mastectomy in 4 (4.045) and 13 (11.6%) patients were not operated.

**Table I:** Result showing Clinical presentation, diagnosis and management of LABC in LMIC.

Characteristics	n =112(%)
***Age in years***	
Mean	52
Range	26-78
***Presentation***	
Size of tumor 5cm & more	103 (91.9%)
Skin involvement	77(68.7%)
Fungating mass	17(15.2%)
Discharging sinus	14(12.5%)
Nipple excoriation	19(16.9%)
Axilla involvement	86(76.7%)
Chest wall involvement	22(19.6%)
T3	71(63.3%)
T4	41(36.6%)
***Diagnosis***	
Histopathology	112 (100%)
Neoadjuvant Chemotherapy	112 (100%)
***ER/PR Status***	
ER and/or PR positive	46(41.1%)
ER and/or PR negative	66(58.9%)
***HER-2/neu status***	
Positive	31(27.7%)
Negative	81(72.3%)
***Staging***	
Non-metastatic	99(88.4%)
Metastatic	13(11.6%)
Liver	4(3.5%)
Lung	3(2.7%)
Bone	6(5.3%)
***Surgery***	
Breast conservation	6(6.1%)
Modified radical mastectomy	86 (86.9%)
Radical Mastectomy	3(3.03%)
Toilet Mastectomy	4(4.04%)
Not operated	13(11.6%)

## DISCUSSION

Our study supports the literature that patient’s delay (delay between individuals’ first awareness of breast abnormality and initial medical consultation) is a common factor that contributes to late detection of breast cancer and presentation at any healthcare facility. It is believed that around 20–30% of women with symptoms of breast cancer wait three months before consulting their physicians.^8^ This is explained by many facts; as it has also been shown that even cells from in situ carcinoma can metastasize.^9^ In the USA, breast cancer death rates decreased by 36% between 1989 and 2012.^10^ These improvements were driven by combined improvements in early detection and adjuvant systemic therapy.^11^ The implementation of screening and treatment policies for breast cancer control that have shown success in high-income settings might be beyond the reach of many countries. The rates of early detection and improved survival has been observed in at upper middle–income countries (UMICs).^12^ The common occurrence of late presentation and advanced stage in LMICs compared with UMICs^13^ contributes to morbidity, a substantial loss of productive life for patients and productive capacity within the community, and, ultimately, to increased breast cancer mortality.^13^,^14^ Data that support the benefits of early detection stems from UMICs,^13^,^15^ where resources are limitless. Thus, only one in seven women (15%) in UMICs presents with locally advanced or metastatic breast cancer.^16^ This is in contrast to LMICs, where nearly two of three women have locally advanced or metastatic disease at the time of presentation.^13^

Our series describes that 65% of our breast cancers are locally advanced at presentation. In developing world late presentation due to mainly social factors leads to compromised outcome for the patients. As opposed to the Western societies where majority of patients are presenting with early cancers, our numbers are telling the story of high morbidity and morbidity.

In absence of National screening programs and widely available Mammography due to lack of resources leads to delayed pick up of disease relying on patient’s self-pick up of the disease. Ironically recent studies show breast physical examination and breast self-examination to be unhelpful in reducing stage at diagnosis^17^ which we used to advocate to our patients initially.

It is also seen that effective therapy may help lower breast cancer death rates after detection^18^,^19^ but still mortality remains high. Neoadjuvant chemotherapy is recommended for women with LABC. Chemotherapy recommendations according to national resources have also been published by the Breast Health Global Initiative.^20^

National diagnostic and treatment guidelines are needed to establish a “standard of care” and promote the rational use of existing resources and greater equity in access to treatment, as stated by WHO.^21^ It cannot be stressed enough that educating women is suggested in all countries to help achieve early detection and treatment.^22^ Evidence-based guidelines outlining optimal approaches to breast cancer detection, diagnosis and treatment have been well developed and disseminated in several high-resource countries.^23^ Breast imaging, initially with ultrasound and, at higher resource levels, with diagnostic mammography, improves pre-operative diagnostic assessment, and also permits image-guided needle sampling of suspicious lesions. Diagnostic mammography and magnetic resonance imaging (MRI), while helpful for breast-conserving surgery, are not mandatory in LMCs when these resources are lacking.^24^The ability to perform modified radical mastectomy (MRM) is the mainstay of locoregional treatment at the basic level of breast healthcare.^25^

## CONCLUSION

In our series 65% of all breast cancers are LABC at presentation. In low/middle income countries high percentage of LABC at presentation result in high metastatic disease, poor prognosis and limits conservation of breast. Awareness and education about breast cancer can have long term impact to reduce the suffering and improve outcomes.

### Author`s Contribution:

**SM** did data collection and manuscript writing.

**MA** conceived, designed and did statistical analysis & editing of manuscript.

**SS** did review and final approval of manuscript.

**DK** did data collection.
